# Supplementary material for: The human LIN28B nucleosome is inherently pre-positioned for efficient binding of multiple OCT4s without H3 K27 acetylation
Source: bioRxiv. 2023 Oct 10:2023.10.06.559923. Preprint. [Version 1] doi: 10.1101/2023.10.06.559923 (PMC10659430; doi:10.1101/2023.10.06.559923)
Supplement: 1 [file NIHPP2023.10.06.559923V1-supplement-1.pdf]

bioRxiv preprint doi: https://doi.org/10.1101/2023.10.06.559923; this version posted October 10, 2023. The copyright holder for this preprint (which was not certified by peer review) is the author/funder. This article is a US Government work. It is not subject to copyright under 17 USC 105 and is also made available for use under a CC0 license.

# Supplemental Methods

## Expression and purification of human core histones

Recombinant human core histones H2A, H2B, H3, and H4 were expressed individually in E. coli BL21(DE3) cells as described in a previous study. Briefly, E. coli cells harboring each histone expression plasmid were grown at 37 °C in 2 x YTB Broth. 0.3 mM IPTG was added to induce recombinant protein expression for 3 h at 37 C, When OD600 reached around 0.6–0.8. The cells were harvested and resuspended in 50 mL of buffer A (50 mM Tris-HCl, 500 mM NaCl, 1 mM PMSF, 5% glycerol, pH 8.0), followed by sonication on ice for 60 min. The cell lysates were centrifuged at 20,000 x *g* for 20 min at 4 °C. The pellet containing histones was resuspended in 50 mL of buffer A and 7M guanidine hydrochloride. The samples were rotated for 12 h, and the supernatant was recovered by centrifugation at 96,000 x *g* for 60 min at 4 °C. The supernatants were dialyzed against buffer C (5 mM Tris-HCl, pH 7.4, 2 mM 2-mercaptoethanol, 7 M urea) for three times. The supernatant was loaded to Hitrap S column chromatography (GE Healthcare). The column was washed with buffer D (20 mM sodium acetate, pH 5.2, 100 mM NaCl, 5 mM 2-mercaptoethanol, 1 mM EDTA, and 6 M urea). The histone protein was eluted with a linear gradient of 100–800 mM NaCl in buffer D. The purified histones were dialyzed against water for three times, and freeze-dried.

## Preparation of 182 bp LIN28B DNA

The 182 bp LIN28B DNA fragment with the same sequence as that used in Sinha et al.'s study was prepared by PCR amplification using the 187 bp LIN28B DNA fragment in Guan et al.' study as the template, followed by ethanol precipitation and purification using the POROS column. The PCR products were pelleted by 75% ethanol containing 0.3 M NaAc at pH 5.2. The sample was

bioRxiv preprint doi: https://doi.org/10.1101/2023.10.06.559923; this version posted October 10, 2023. The copyright holder for this preprint (which was not certified by peer review) is the author/funder. This article is a US Government work. It is not subject to copyright under 17 USC 105 and is also made available for use under a CC0 license.

incubated for 60 min at -20 °C, followed by centrifugation. The pellet was resuspended in TE buffer. The sample was loaded to POROS column chromatography (GE Healthcare). The column was washed with buffer containing 20 mM Tris-HCl, pH 7.4, 5 mM 2-mercaptoethanol, and the DNA was eluted by a linear gradient of 0–2 M NaCl.

**Reconstitution of the 182 bp LIN28B nucleosome with human core histones**

Purified recombinant human core histones in equal stoichiometric ratio were dissolved in 6 mL unfolding buffer (7 M guanidine-HCl, 20 mM Tris-Cl at pH 7.4, 10 mM DTT) and subsequently dialyzed against refolding buffer (10 mM Tris-Cl at pH 7.4, 1 mM EDTA, 5 mM b-mercaptoethanol, 2 M NaCl) for about 12 h. The mixture was centrifuged at 20,000 x *g* to remove any insoluble material. Soluble octamers were purified by size fractionation on a Superdex 200 gel filtration column. Nucleosome assembly was done by following the 'double bag' dialysis method of Sinha et al. Briefly, the histone octamer and the 182 bp DNA fragment were mixed in equimolar ratios in high salt buffer containing 25 mM HEPES (pH 7.5), 2 M NaCl and 2 mM DTT. The mixture was filled into a 3.5kDa cut-off dialysis bag and dialyzed against the high salt buffer for 3 hours. The dialysis bag was then transferred into 1L of the buffer containing 25 mM HEPES (pH 7.5), 1 M NaCl and 1 mM DTT for overnight dialysis. The buffer was changed to 25 mM HEPES (pH 7.5) and 1 mM DTT for the second overnight dialysis. The reconstituted nucleosome was stored at 4 °C for the EMSA study.

**Electrophoretic assays and band intensity measurement**

The reconstituted nucleosome samples were analyzed using native polyacrylamide gel electrophoresis (4%) in 0.2 x TBE at 120 V for 70 min at 4 °C. After electrophoresis, the gel was

bioRxiv preprint doi: https://doi.org/10.1101/2023.10.06.559923; this version posted October 10, 2023. The copyright holder for this preprint (which was not certified by peer review) is the author/funder. This article is a US Government work. It is not subject to copyright under 17 USC 105 and is also made available for use under a CC0 license.

stained with ethidium bromide (EtBr). To investigate the histone components of the bands in the native gel, the bands were cut out and soaked in 2x SDS loading buffer. After heating at 90 ºC for 10 mins, the samples were loaded to the SDS page gel. Band intensities in the gels were quantified using ImageJ.

bioRxiv preprint doi: https://doi.org/10.1101/2023.10.06.559923; this version posted October 10, 2023. The copyright holder for this preprint (which was not certified by peer review) is the author/funder. This article is a US Government work. It is not subject to copyright under 17 USC 105 and is also made available for use under a CC0 license.

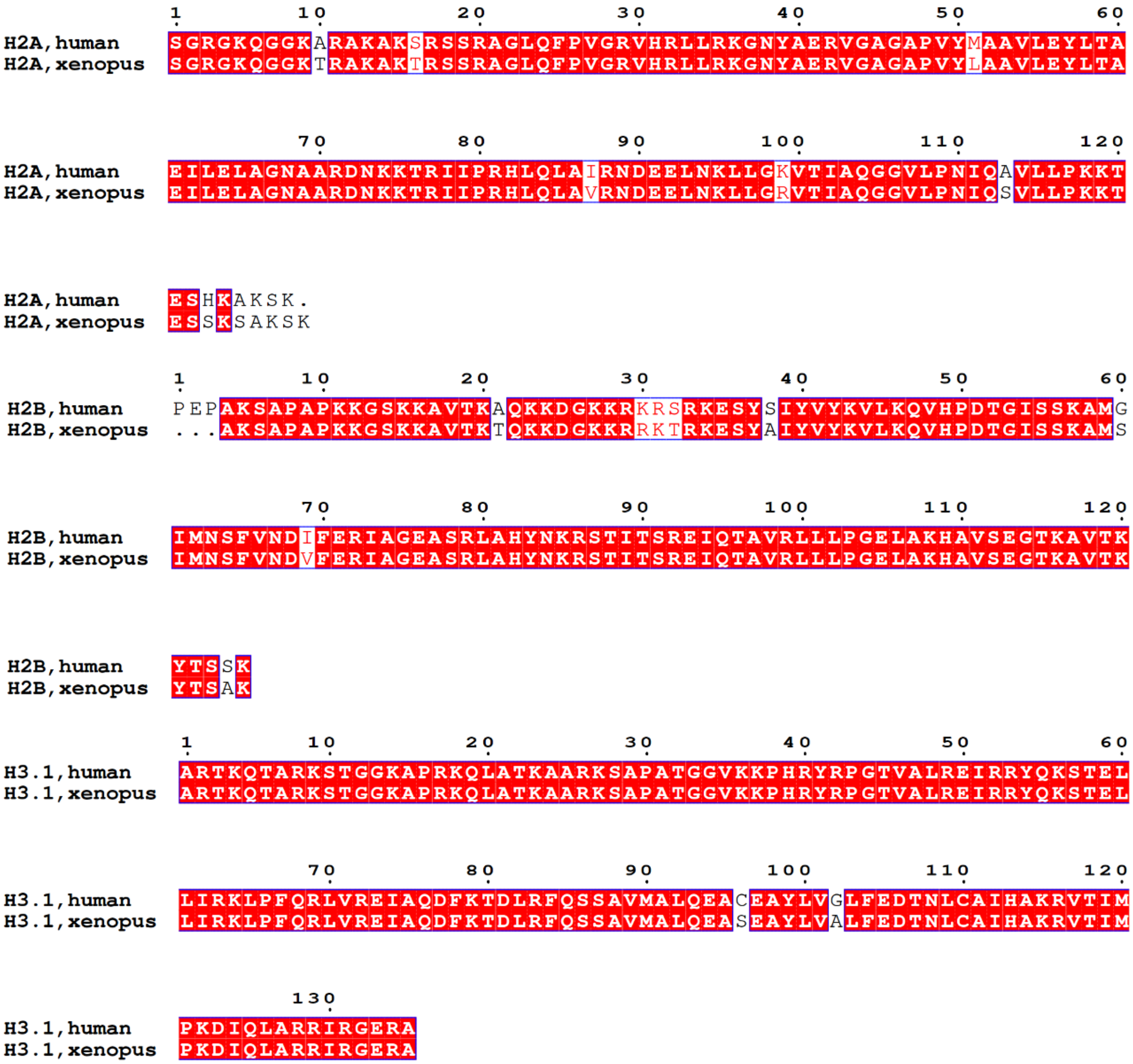

**Supplementary Fig. 1 Comparison of human and *Xenopus larvis* core histone sequences.**

bioRxiv preprint doi: https://doi.org/10.1101/2023.10.06.559923; this version posted October 10, 2023. The copyright holder for this preprint (which was not certified by peer review) is the author/funder. This article is a US Government work. It is not subject to copyright under 17 USC 105 and is also made available for use under a CC0 license.

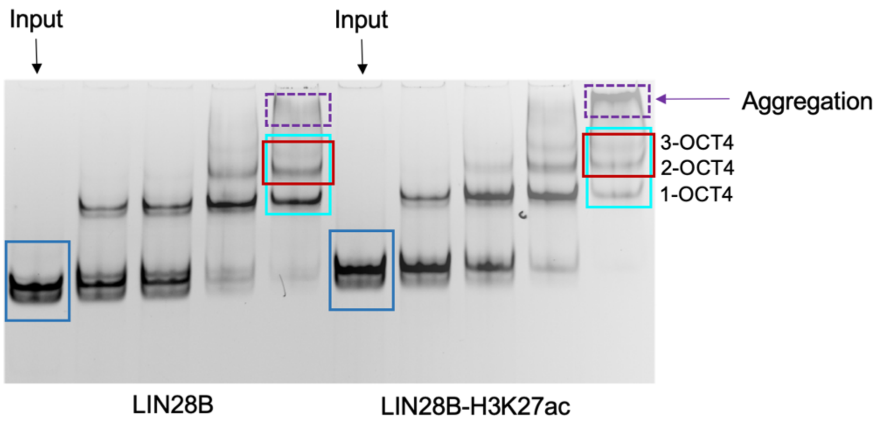


| | LIN28B | LIN28B H3K27ac |
|---|---|---|
| 2-OCT4 + 3-OCT4 | 30.1% | 25.1% |
| 1-OCT4 + 2-OCT4 + 3-OCT4 | 76.7% | 49.8% |
| Aggregation | 18.8% | 38.7% |

**Supplementary Fig. 2. H3 K27 acetylation does not increase the population of the nucleosome bound to two and three OCT4s versus input.** Illustration of band intensity measurement of OCT4 binding to the LIN28B nucleosome using Sinha et al.'s gel (Fig. 3**b** in Sinha et al.[1]) and ImageJ (upper panel; the boxes indicate the area used for intensity measurement), and percentage of the intensity ratio of the nucleosome bound to OCT4 over the input.
